# Association of Parity and Breastfeeding With Risk of Early Natural Menopause

**DOI:** 10.1001/jamanetworkopen.2019.19615

**Published:** 2020-01-22

**Authors:** Christine R. Langton, Brian W. Whitcomb, Alexandra C. Purdue-Smithe, Lynnette L. Sievert, Susan E. Hankinson, JoAnn E. Manson, Bernard A. Rosner, Elizabeth R. Bertone-Johnson

**Affiliations:** 1 School of Public Health and Health Sciences, Department of Biostatistics and Epidemiology, University of Massachusetts, Amherst; 2 Epidemiology Branch, Division of Intramural Population Health Research, *Eunice Kennedy Shriver* National Institute of Child Health and Human Development, Bethesda, Maryland; 3 Department of Anthropology, University of Massachusetts, Amherst; 4 Channing Division of Network Medicine, Department of Medicine, Brigham and Women’s Hospital and Harvard Medical School, Boston, Massachusetts; 5 Department of Epidemiology, Harvard T.H. Chan School of Public Health, Boston, Massachusetts; 6 Division of Preventive Medicine, Brigham and Women’s Hospital and Harvard Medical School, Boston, Massachusetts; 7 Department of Biostatistics, Harvard T.H. Chan School of Public Health, Boston, Massachusetts; 8 School of Public Health and Health Sciences, Department of Health Promotion and Policy, University of Massachusetts, Amherst

## Abstract

**Importance:**

Pregnancy and breastfeeding prevent ovulation and may slow the depletion of the ovarian follicle pool. These factors may lower the risk of early menopause, a condition associated with increased risk of cardiovascular disease and other adverse health outcomes.

**Objective:**

To examine the association of parity and breastfeeding with the risk of early menopause.

**Design, Setting, and Participants:**

This population-based cohort study within the Nurses’ Health Study II cohort (1989-2015) included premenopausal participants who were aged 25 to 42 years at baseline. Response rates were 85% to 90% for each cycle, and follow-up continued until menopause, age 45 years, hysterectomy, oophorectomy, death, cancer diagnosis, loss to follow-up, or end of follow-up in May 2015. Hypotheses were formulated after data collection. Data analysis took place from February to July 2019.

**Exposures:**

Parity (ie, number of pregnancies lasting ≥6 months) was measured at baseline and every 2 years. History and duration of total and exclusive breastfeeding were assessed 3 times during follow-up. Menopause status and age were assessed every 2 years.

**Main Outcomes and Measures:**

Risk of natural menopause before age 45 years.

**Results:**

At baseline, 108 887 premenopausal women aged 25 to 42 years (mean [SD] age, 34.1 [4.6] years; 102 246 [93.9%] non-Hispanic white) were included in the study. In multivariable models, higher parity was associated with lower risk of early menopause. Hazard ratios were attenuated with adjustment for breastfeeding but remained significant. Compared with nulliparous women, those reporting 1, 2, 3, and 4 or more pregnancies lasting at least 6 months had hazard ratios for early menopause of 0.92 (95% CI, 0.79-1.06), 0.84 (95% CI, 0.73-0.96), 0.78 (95% CI, 0.67-0.92), and 0.81 (95% CI, 0.66-1.01), respectively (*P *for trend = .006). In multivariable models also adjusted for parity, hazard ratios for duration of exclusive breastfeeding of 1 to 6, 7 to 12, 13 to 18, and 19 or more months were 0.95 (95% CI, 0.85-1.07), 0.72 (95% CI, 0.62-0.83), 0.80 (95% CI, 0.66-0.97), and 0.89 (95% CI, 0.69-1.16), respectively, compared with less than 1 month of exclusive breastfeeding (*P *for trend = .001). Despite the significant test for trend, estimates were not observed to be lower as duration of exclusive breastfeeding increased. In a stratified analysis of parous women, risk of early menopause was lowest among those reporting exclusive breastfeeding for 7 to 12 months in each level of parity (women with 2 pregnancies and 7-12 months of breastfeeding: HR, 0.79; 95% CI, 0.66-0.96; ≥3 pregnancies and 7-12 months of breastfeeding: HR, 0.68; 95% CI, 0.52-0.88; 2 pregnancies and ≥13 months of breastfeeding: HR, 0.87; 95% CI, 0.66-1.15; ≥3 pregnancies and 13-18 months of breastfeeding: HR, 0.86; 95% CI, 0.66-1.13; and ≥3 pregnancies and ≥19 months of breastfeeding: HR, 0.98; 95% CI, 0.72-1.32).

**Conclusions and Relevance:**

In this study, an inverse association of parity with risk of early menopause was observed. Breastfeeding was associated with significantly lower risk, even after accounting for parity. Breastfeeding at levels consistent with current recommendations may confer an additional benefit of lower risk of early menopause.

## Key Points

**Question:**

What is the association of parity and breastfeeding with early natural menopause?

**Findings:**

In this cohort study that included 108 887 premenopausal women, parity and breastfeeding were each associated with a significantly lower risk of early natural menopause. Findings for breastfeeding suggest that some of the lower risk attributed to parity could be attributable to breastfeeding.

**Meaning:**

The findings suggest that breastfeeding at levels consistent with current recommendations may confer an additional benefit of lower risk of early menopause.

## Teaser

This cohort study examines the association of parity and breastfeeding with the risk of early natural menopause among women in the Nurses’ Health Study II cohort.

## Introduction

Early menopause, defined as the cessation of ovarian function before the age of 45 years, affects approximately 10% of women in Western populations.^1^ Current research suggests that women who experience early menopause are at increased risk of premature mortality, cognitive decline, osteoporosis, and cardiovascular disease.^2,3^

The reproductive aging process is characterized by the gradual decrease in both quantity and quality of oocytes within ovarian follicles.^1^ The number of oocytes a woman is born with, the rate of loss of those oocytes during the life span because of the process of atresia, and the threshold number of oocytes needed to produce sufficient hormones to maintain menstrual cyclicity have been identified as determinants of age at menopause.^1,4,5,6^ Reproductive events that interrupt ovulation or modify the rate of follicular atresia, including pregnancy and breastfeeding, slow the depletion of the ovarian follicle pool and are hypothesized to be associated with delayed menopause.^7,8,9^

While the association of reproductive factors with the timing of menopause has been previously examined in the literature,^8,10,11,12,13,14,15,16,17,18,19,20,21,22,23,24,25,26,27,28,29,30,31^ few studies have specifically examined the risk of early menopause as an outcome.^32^ A study by Mishra et al^32^ examined the association of parity with risk of menopause at several age categories and observed a significant inverse association for women who were younger than 40 years, aged 40 to 44 years, and aged 45 to 49 years at menopause, similar to other studies examining parity and menopause timing.^8,12,18,21,22,23,26,33^ A small number of cross-sectional and case-control studies have examined the association of breastfeeding with menopause timing,^16,17,18,21,30,34,35^ and as such, important questions remain. Furthermore, most of these studies have been limited by cross-sectional design and retrospective recall of reproductive factors and menopause timing. If age-related declines in viable oocytes lead to lower parity because of lower fecundity as well as earlier timing of menopause, results from these analyses may be biased because of reverse causality.^1^ Prospective studies that account for both the timing of reproductive events and the onset of menopause are needed to disentangle these complicated questions. We therefore aimed to examine the association of parity and breastfeeding with early natural menopause in the prospective Nurses’ Health Study II (NHS2).

## Methods

The NHS2 is a prospective cohort study that began in June 1989 when 116 429 female registered nurses aged 25 to 42 years from 14 US states responded to a baseline questionnaire. At baseline, participants provided information regarding their medical history and health-related behaviors, such as use of oral contraceptives (OCs), menstrual and pregnancy history, and smoking status. Cohort members completed questionnaires every 2 years to identify new diagnoses of disease and update information on health-related behaviors. Questionnaire response rates were 85% to 90% for each cycle.^36^ The study protocol was approved by the institutional review boards at Brigham and Women’s Hospital and the Harvard T.H. Chan School of Public Health, and participants provided written informed consent. This study followed the Strengthening the Reporting of Observational Studies in Epidemiology (STROBE) reporting guideline for cohort studies.

### Assessment of Reproductive Factors

Parity was measured at baseline in 1989 and then updated every 2 years to 2009 by asking women to report whether they had been pregnant within the past 2 years and the number of pregnancies lasting 6 months or longer. Complete pregnancy history was verified in 2009 by asking participants to report the calendar year each pregnancy ended. Age at first birth was first reported at baseline and, for first pregnancies after 1989, was derived from the woman’s age and timing of births reported on biennial questionnaires.

Breastfeeding history was first measured in 1993 by asking participants how many months in total they breastfed for all their births combined. Women were also able to indicate if they did not breastfeed, had no children, or could not remember. In 1997, women who had breastfed for at least 1 month were asked when they started to give formula or purchased milk at least once daily, started giving solid food at least once daily, started pumping breast milk at least 4 days per week, and went at least 6 hours at night without breastfeeding. Information on each pregnancy was collected for women reporting breastfeeding for up to 4 children. Women who breastfed more than 4 children were asked to indicate the total number of months they breastfed all other children combined. Women reporting pregnancies after 1997 provided similar detailed information in 2003. Responses to these questions allowed for the derivation of 2 breastfeeding variables, as follows: cumulative exclusive breastfeeding, defined as the period of time during which women fed their infants breast milk only with no other liquids or solids provided, and cumulative total breastfeeding, which includes all breastfeeding, both exclusive and nonexclusive.

### Assessment of Early Menopause

Beginning in 1989, NHS2 members were asked if their menstrual periods had ceased permanently with the following response options: “no: premenopausal,” “yes: no menstrual periods,” “yes: had menopause but now have periods induced by hormones,” and “not sure.” Women reporting that their periods had ceased were then asked, “Age periods ceased?” with an open response field, and “For what reason did your periods cease?” with response options of surgery, radiation or chemotherapy, or natural. Women were also asked about use of hormone therapy (HT), including timing and type used. These questions were repeated on each biennial questionnaire. Age at menopause was defined as age after 12 consecutive months of amenorrhea. For the women who reported being postmenopausal on 1 questionnaire and then subsequently reported being premenopausal, we defined age at menopause as the age at which periods ceased for at least 12 months, followed by consistent reporting of cessation on at least 3 consecutive questionnaires.

The present study was limited to 108 887 women who were premenopausal at baseline, had no prior cancer diagnosis (except for nonmelanoma skin cancer), and had no history of hysterectomy or oophorectomy. We defined cases of early menopause as women who reported natural menopause at younger than 45 years.

### Covariates

Age at menarche, race/ethnicity, and height were reported in 1989. Body mass index (calculated as weight in kilograms divided by height in meters squared) was calculated using height reported at baseline and weight reported biennially. Age, smoking status, number of cigarettes smoked per day, OC use, and duration of OC use were assessed every 2 years. Infertility was assessed at baseline by asking women if they had ever tried to become pregnant for more than 1 year without success and, if so, what the cause was (eg, ovulatory disorder, tubal blockage, spouse or partner). On biennial questionnaires to 2001 and every 4 years thereafter, women were asked if they experienced infertility since the last questionnaire. Dietary factors, including percentage of vegetable protein intake, amount of vitamin D from foods and supplements, and alcohol intake, were assessed in 1991 and every 4 years thereafter via semiquantitative food frequency questionnaires. Intake measures were adjusted for energy using the residual method.^37^

### Statistical Analysis

Baseline characteristics were examined according to baseline measures of parity and duration of exclusive breastfeeding using generalized linear models. We used Cox proportional hazards models to estimate age-adjusted hazard ratios (HRs) and 95% CIs for the association of parity and breastfeeding with early menopause. Participants contributed follow-up time from the return of the baseline questionnaire in 1989 until the onset of menopause; age 45 years; first report of a hysterectomy, bilateral or unilateral oophorectomy, or cancer (not including nonmelanoma skin cancer); death; loss to follow-up; or the end of follow-up in May 2015, whichever came first. Analyses were stratified by age and questionnaire cycle. Linear trends were assessed and tested by modeling parity and age at first birth as continuous variables and category medians for duration of total and exclusive breastfeeding.

Models of early menopause risk examined the timing of reproductive events by updating data as they became available from the biennial questionnaires. We ran models updating parity every 2 years to assess the association of risk with current parity. To assess associations with cumulative duration of total and exclusive breastfeeding, updated information was available through 2003.

To control for potential confounding, we created 2 multivariable models: model 1, adjusted for age; and model 2, adjusted for age, age at menarche (ie, ≤9, 10, 11, 12, 13, 14, 15, 16, or ≥17 years), smoking status (ie, never, past [1-14, 15-24, or ≥25 years], or current [1-14, 15-24, or ≥25 years]), alcohol intake (ie, 0, 1 to <10, 10 to <30, or ≥30 g/d), vegetable protein intake (in quintiles), body mass index (ie, <18.5, 18.5-24.9, 25.0-29.9, or ≥30), vitamin D from dairy foods (in quintiles), supplemental vitamin D (ie, 0, >0 to <600, or ≥600 IU/d), OC use (ie, never, past, or current), duration of OC (ie, never, 1-23, 24-47, 48-71, 72-95, 96-119, ≥120 months, or missing), and infertility because of ovulatory disorder (ie, yes or no). Covariates were identified a priori from previous literature and associations with early menopause in the NHS2 population. We included infertility because of ovulatory disorder in our multivariable models to account for heterogeneity among the nulliparous women in our data set. Additional covariates were considered for inclusion in multivariable models (eg, physical activity, race/ethnicity, age at last birth), but none were observed to substantively affect exposure estimates or model fit. We first evaluated each factor in models that did not control for the other reproductive factor. To assess the independent associations of parity and breastfeeding with risk of early menopause, the reproductive factors were mutually adjusted in our final models (ie, model 3).

To examine whether the association of breastfeeding with risk of early menopause differed by level of parity, we ran models stratified by parity (ie, 1, 2, or ≥3 pregnancies). We investigated the potential variation in associations by the timing of reproductive events by evaluating risk of early menopause by number of pregnancies by the ages of 25, 30, and 35 years. To minimize potential confounding by infertility, we conducted a sensitivity analysis restricted to women without a history of self-reported infertility. To evaluate whether HT use affected associations, we conducted 2 additional sensitivity analyses, as follows: (1) adjusting for HT use and (2) censoring women at first report of HT use.

Clean and complete data used for this analysis were available in February 2019, and analyses were conducted from February through July 2019. All statistical analyses were conducted with SAS version 9.4 (SAS Institute). Statistical significance was set at a 2-tailed *P* < .05 for all analyses.

## Results

Characteristics by categories of parity for 108 887 premenopausal participants aged 25 to 42 years at baseline (mean [SD] age, 34.1 [4.6] years; 102 246 [93.9%] non-Hispanic white) are shown in Table 1. At baseline, the prevalence of past or current smoking was lowest among women with 4 or more pregnancies (1460 of 4528 [32.2%] vs 11 143 of 33 110 [33.7%] with 0 pregnancies vs 7638 of 21 013 [36.3%] with 1 pregnancy vs 12 235 of 35 108 [34.8%] with 2 pregnancies vs 5029 of 15 128 [33.2%] with 3 pregnancies). Women with 1 pregnancy had the highest prevalence of past or current OC use (18 199 [86.6%] vs 25 618 [77.3%] with 0 pregnancies vs 30 309 [86.3%] with 2 pregnancies vs 12 602 [83.3%] with 3 pregnancies vs 3502 [77.3%] with ≥4 pregnancies) and infertility (4833 [23.0%] vs 5099 [15.4%] with 0 pregnancies vs 6144 [17.5%] with 2 pregnancies vs 2466 [16.3%] with 3 pregnancies vs 679 [15.0%] with ≥4 pregnancies). Mean (SD) cumulative duration of total breastfeeding was positively associated with higher parity (women with 1 pregnancy, 6.0 [6.0] months; 2 pregnancies, 11.9 [10.3] months; 3 pregnancies, 19.7 [14.9] months; and ≥4 pregnancies, 29.8 [20.9] months).

**Table 1.** Baseline Characteristics of 108 887 Participants by Parity in the Nurses’ Health Study II, 1989-2015^a^

Characteristic	Mean (SD) by No. of Pregnancies				
	0 (n = 33 110)	1 (n = 21 013)	2 (n = 35 108)	3 (n = 15 128)	≥4 (n = 4528)
Age, y	32.3 (4.8)	33.3 (4.6)	35.2 (4.2)	35.9 (3.8)	36.9 (3.6)
Smoking, pack-years^b^	18.1 (84.8)	17.5 (80.1)	18.4 (83.9)	16.5 (70.0)	19.5 (85.9)
Body mass index^c^	23.8 (5.3)	24.0 (5.0)	24.0 (4.8)	24.2 (4.7)	24.5 (4.9)
Physical activity, MET-h/wk	34.2 (68.2)	27.1 (65.7)	26.2 (73.1)	24.6 (67.1)	24.3 (66.2)
Oral contraceptive use, mo^d^	86.5 (152.5)	77.1 (134.4)	69.6 (133.3)	66.4 (151.6)	67.0 (172.4)
Vegetable protein intake, % total, kcal	5.0 (1.2)	5.0 (1.1)	5.0 (1.1)	5.0 (1.0)	5.0 (1.0)
Calcium intake, mg/d	1046 (457.1)	1041 (436.9)	994 (417.7)	981 (400.0)	992 (405.3)
Vitamin D intake, IU/d	425 (286.2)	415 (266.5)	364 (245.4)	347 (229.3)	354 (252.2)
Alcohol intake, g/d	4.0 (6.8)	2.7 (5.3)	2.6 (5.3)	2.3 (4.5)	1.9 (4.2)
Non-Hispanic white, No. (%)	30 759 (92.9)	19 542 (93.0)	33 247 (94.7)	14 387 (95.1)	4311 (95.2)
Smoking status, No. (%)					
Past	6249 (18.9)	4742 (22.6)	7958 (22.7)	3292 (21.8)	954 (21.1)
Current	4894 (14.8)	2896 (13.8)	4277 (12.2)	1737 (11.5)	506 (11.2)
Age at menarche, y	12.4 (1.5)	12.4 (1.4)	12.4 (1.4)	12.4 (1.4)	12.5 (1.4)
Oral contraceptive use, No. (%)					
Past	18 577 (56.2)	15 575 (74.2)	27 468 (78.3)	11 967 (79.2)	3389 (75.1)
Current	7041 (21.3)	2624 (12.5)	2841 (8.1)	635 (4.2)	113 (2.5)
History of infertility, No. (%)	5099 (15.4)	4833 (23.0)	6144 (17.5)	2466 (16.3)	679 (15.0)
Age at first birth, y	NA	27.5 (4.4)	25.5 (3.7)	24.2 (3.3)	23.1 (3.2)
Total breastfeeding duration, mo^e^	NA	6.0 (6.0)	11.9 (10.3)	19.7 (14.9)	29.8 (20.9)
Exclusive breastfeeding duration, mo^e^	NA	2.2 (2.7)	4.4 (4.8)	7.2 (6.9)	10.7 (9.4)

Abbreviations: MET-h, metabolic equivalent of hours; NA, not applicable.

^a^ Parity was defined as the number of pregnancies lasting at least 6 months.

^b^ Among past or current smokers.

^c^ Calculated as weight in kilograms divided by height in meters squared.

^d^ Among past or current users of oral contraceptives.

^e^ Among parous women.

Baseline characteristics of 59 388 parous women by duration of exclusive breastfeeding are shown in Table 2. Women who breastfed for 19 or more cumulative months had the lowest rates of OC use (1544 of 2061 [74.9%] vs 22 031 of 25 228 [87.3%] who breastfed for <1 month vs 12 965 of 15 008 [86.4%] who breastfed for 1-6 months vs 10 189 of 12 168 [83.7%] who breastfed for 7-12 months vs 3954 of 4923 [80.3%] who breastfed for 13-18 months), smoking (508 [24.6%] vs 9347 [37.1%] who breastfed for <1 month vs 5036 [33.6%] who breastfed for 1-6 months vs 3828 [31.5%] who breastfed for 7-12 months vs 1341 [27.2%] who breastfed for 13-18 months), and mean (SD) alcohol intake (1.9 [4.2] g/d vs 2.6 [5.2] g/d among women who breastfed for <1 month vs 2.6 [5.1] g/d among women who breastfed for 1-6 months vs 2.5 [4.8] g/d among women who breastfed for 7-12 months vs 2.1 [4.1] g/d among women who breastfed for 13-18 months). Higher mean (SD) parity was associated with longer duration of breastfeeding (<1 month, 1.9 [0.8]; 1-6 months, 1.8 [0.8]; 7-12 months, 2.2 [0.8]; 13-18 months, 2.7 [0.8]; ≥19 months, 3.3 [0.9]).

**Table 2.** Baseline Characteristics of 59 388 Parous Women With Breastfeeding Data by Duration of Cumulative Exclusive Breastfeeding in the Nurses’ Health Study II, 1989-2015^a^

Characteristic	Mean (SD) by Breastfeeding Duration, mo				
	<1 (n = 25 228)	1-6 (n = 15 008)	7-12 (n = 12 168)	13-18 (n = 4923)	≥19 (n = 2061)
Age, y	35.2 (4.6)	34.4 (4.4)	35.0 (4.1)	35.5 (3.6)	36.0 (3.6)
Smoking, pack-years^b^	18.2 (76.6)	15.9 (72.5)	16.4 (73.4)	17.1 (85.4)	15.5 (76.3)
Body mass index^c^	24.5 (5.1)	23.8 (4.5)	23.6 (4.4)	23.5 (4.3)	23.6 (4.3)
Physical activity, MET-h/wk	24.3 (64.7)	26.3 (73.2)	26.1 (68.1)	24.8 (59.2)	26.9 (68.7)
Oral contraceptive use, mo^d^	72.9 (134.8)	71.6 (135.9)	65.0 (131.9)	61.6 (139.5)	61.4 (149.9)
Vegetable protein intake, % total kcal	4.9 (1.0)	5.0 (1.1)	5.1 (1.1)	5.2 (1.1)	5.3 (1.2)
Calcium intake, mg/d	967 (416.4)	1023 (414.1)	1051 (419.0)	1057 (414.6)	1068 (422.4)
Vitamin D intake, IU/d	359 (251.0)	386 (245.3)	388 (243.1)	383 (241.7)	385 (252.8)
Alcohol intake, g/d	2.6 (5.2)	2.6 (5.1)	2.5 (4.8)	2.1 (4.1)	1.9 (4.2)
Non-Hispanic white, No. (%)	23 840 (94.5)	14 273 (95.1)	11 706 (96.2)	4756 (96.6)	1991 (96.6)
Smoking status, No. (%)					
Past	5593 (22.2)	3402 (22.7)	2844 (23.4)	1056 (21.5)	403 (19.6)
Current	3754 (14.9)	1634 (10.9)	984 (8.1)	285 (5.8)	105 (5.1)
Age at menarche, y	12.4 (1.4)	12.4 (1.4)	12.4 (1.4)	12.5 (1.4)	12.5 (1.4)
Oral contraceptive use, No. (%)					
Past	19 561 (77.6)	11 676 (77.9)	9423 (77.5)	3762 (76.5)	1491 (72.6)
Current	2470 (9.8)	1289 (8.6)	766 (6.3)	192 (3.9)	53 (2.6)
History of infertility, No. (%)	4819 (19.1)	2942 (19.6)	2227 (18.3)	832 (16.9)	359 (17.4)
Age at first birth, y	25.2 (4.2)	26.3 (4.0)	26.1 (3.7)	25.6 (3.3)	25.2 (3.2)
Parity	1.9 (0.84)	1.8 (0.81)	2.2 (0.83)	2.7 (0.81)	3.3 (0.93)
Total breastfeeding duration, mo	4.1 (6.8)	11.9 (7.5)	20.2 (9.3)	31.0 (11.0)	43.6 (14.1)

Abbreviation: MET-h, metabolic equivalent of hours.

^a^ Nulliparous women (n = 33 110) and women with missing breastfeeding data (n = 16 389) are not shown. Exclusive breastfeeding is when infant received breast milk only, and no other liquids or solids were given.

^b^ Among past or current smokers.

^c^ Calculated as weight in kilograms divided by height in meters squared.

^d^ Among past or current users of oral contraceptives.

During 1.7 million person-years of follow-up, 2571 women (2.4%) in the cohort experienced early natural menopause. In both age-adjusted and multivariable-adjusted models, higher parity was associated with lower risk of early menopause (model 1: 1 pregnancy, HR, 0.87; 95% CI, 0.77-0.99; 2 pregnancies, HR, 0.78; 95% CI, 0.70-0.86; 3 pregnancies, HR, 0.70; 95% CI, 0.62-0.79; ≥4 pregnancies, HR, 0.69; 95% CI, 0.58-0.83; *P *for trend < .001; model 2: 1 pregnancy, HR, 0.86; 95% CI, 0.76-0.97; 2 pregnancies, HR, 0.77; 95% CI, 0.69-0.85; 3 pregnancies, HR, 0.70; 95% CI, 0.61-0.79; ≥4 pregnancies, HR, 0.70; 95% CI, 0.59-0.84; *P *for trend < .001) (Table 3). Adjustment for covariates, including infertility because of ovulatory disorder, minimally affected the association of parity with early menopause. After controlling for breastfeeding, estimates were attenuated but followed the same pattern of decreasing risk with increasing parity. Compared with nulliparous women, those reporting 1, 2, 3, and 4 or more births had HRs for early menopause of 0.92 (95% CI, 0.79-1.06), 0.84 (95% CI, 0.73-0.96), 0.78 (95% CI, 0.67-0.92), and 0.81 (95% CI, 0.66-1.01), respectively (*P *for trend = .006). In analyses limited to women without a history of infertility, results were slightly stronger; for example, the HR among women with 4 or more pregnancies was 0.77 (95% CI, 0.61-0.97; *P* for trend = .005) (data not shown).

**Table 3.** Multivariable Associations of Parity With Early Menopause in the Nurses’ Health Study II, 1989-2015^a^

Parity	Cases, No.	Person-Years	Model 1		Model 2^b^		Model 3^c^	
			HR (95% CI)	*P* Value for Trend	HR (95% CI)	*P* Value for Trend	HR (95% CI)	*P* Value for Trend
0	624	193 733	1 [Reference]	<.001	1 [Reference]	<.001	1 [Reference]	.006
1	423	142 831	0.87 (0.77-0.99)		0.86 (0.76-0.97)		0.92 (0.79-1.06)	
2	929	317 516	0.78 (0.70-0.86)		0.77 (0.69-0.85)		0.84 (0.73-0.96)	
3	441	162 176	0.70 (0.62-0.79)		0.70 (0.61-0.79)		0.78 (0.67-0.92)	
≥4	154	56 049	0.69 (0.58-0.83)		0.70 (0.59-0.84)		0.81 (0.66-1.01)	

Abbreviation: HR, hazard ratio.

^a^ Parity was defined as the number of pregnancies lasting at least 6 months.

^b^ Adjusted for age, age at menarche (ie, ≤9, 10, 11, 12, 13, 14, 15, 16, or ≥17 years), smoking status (ie, never, past [1-14, 15-24, or ≥25 years], or current [1-14, 15-24, or ≥25 years]), alcohol intake (ie, 0, 1 to <10, 10 to <30, or ≥30 g/d), vegetable protein intake (in quintiles), body mass index (ie, <18.5, 18.5-24.9, 25.0-29.9, or ≥30), vitamin D from dairy foods (in quintiles), supplemental vitamin D (ie, 0, >0 to <600, or ≥600 IU/d), OC use (ie, never, past, or current), duration of OC (ie, never, 1-23, 24-47, 48-71, 72-95, 96-119, ≥120 months, or missing), and infertility because of ovulatory disorder (ie, yes or no).

^c^ Additionally adjusted for total duration of breastfeeding (ie, <1, 1-3, 4-6, 7-12, 13-18, 19-24, 25-36, or ≥36 months, or parous but breastfeeding data missing).

Longer duration of breastfeeding was associated with lower risk of early menopause (Table 4). In age-adjusted models, women who breastfed for 25 months or longer during their premenopausal years had a significantly lower risk of early menopause compared with women who breastfed for less than 1 month (model 1, HR, 0.73; 95% CI, 0.63-0.85; *P* for trend < .001). Estimates from multivariable models 2 and 3 were slightly attenuated, but followed the same pattern of decreasing risk with increasing duration. For example, after adjusting for all covariates in model 3, those who breastfed for 25 months or longer had an HR of 0.84 (95% CI, 0.71-0.99) compared with women who breastfed for less than 1 month (*P* for trend = .01). Attenuation appeared to be because of the cumulative contributions of all covariates and not a single covariate. Duration of exclusive breastfeeding was also associated with lower risk of early menopause. In fully adjusted models that also accounted for differences in parity (ie, model 3), we observed lower risk among women who breastfed exclusively for 7 to 12 months (HR, 0.72; 95% CI, 0.62-0.83) and 13 to 18 months (HR, 0.80; 95% CI, 0.66-0.97) compared with women who breastfed for less than 1 month (*P* for trend = .001). Women who breastfed for 1 to 6 months had an HR of 0.95 (95% CI, 0.85-1.07), and those who breastfed for 19 or more months had an HR of 0.89 (95% CI, 0.69-1.16).

**Table 4.** Multivariable Associations Between Cumulative Total and Exclusive Breastfeeding and Early Menopause Among Parous Women in the Nurses’ Health Study II, 1989-2015^a^

Breastfeeding	Cases, No.	Person-Years	Model 1		Model 2^b^		Model 3^c^	
			HR (95% CI)	*P* Value for Trend	HR (95% CI)	*P* Value for Trend	HR (95% CI)	*P* Value for Trend
**Cumulative Total Breastfeeding Duration, mo**								
<1	355	95 199	1 [Reference]	<.001	1 [Reference]	<.001	1 [Reference]	.01
1-6	313	92 804	0.99 (0.85-1.16)		1.01 (0.87-1.18)		1.0 (0.86-1.17)	
7-12	331	110 884	0.87 (0.75-1.01)		0.90 (0.77-1.05)		0.90 (0.78-1.05)	
13-18	231	82 977	0.81 (0.68-0.95)		0.85 (0.72-1.0)		0.87 (0.73-1.03)	
19-24	176	61 646	0.80 (0.66-0.95)		0.86 (0.71-1.03)		0.89 (0.74-1.07)	
≥25	335	125 846	0.73 (0.63-0.85)		0.80 (0.68-0.93)		0.84 (0.71-0.99)	
**Cumulative Exclusive Breastfeeding Duration, mo**								
<1	737	202 123	1 [Reference]	<.001	1 [Reference]	<.001	1 [Reference]	.001
1-6	406	126 152	0.93 (0.82-1.05)		0.95 (0.84-1.07)		0.95 (0.84-1.07)	
7-12	280	116 419	0.67 (0.59-0.77)		0.71 (0.61-0.81)		0.72 (0.62-0.83)	
13-18	140	54 207	0.72 (0.60-0.86)		0.77 (0.64-0.92)		0.80 (0.66-0.97)	
≥19	70	24 282	0.77 (0.60-0.98)		0.84 (0.65-1.07)		0.89 (0.69-1.16)	

Abbreviation: HR, hazard ratio.

^a^ Exclusive breastfeeding was defined as when the infant received breast milk only, with no other liquids or solids given. Total breastfeeding includes all breastfeeding, both exclusive and nonexclusive. Data were from 63 357 parous women who provided information on breastfeeding, of whom 59 388 provided information on exclusive breastfeeding.

^b^ Adjusted for age, age at menarche (ie, ≤9, 10, 11, 12, 13, 14, 15, 16, or ≥17 years), smoking status (ie, never, past [1-14, 15-24, or ≥25 years], or current [1-14, 15-24, or ≥25 years]), alcohol intake (ie, 0, 1 to <10, 10 to <30, or ≥30 g/d), vegetable protein intake (in quintiles), body mass index (ie, <18.5, 18.5-24.9, 25.0-29.9, or ≥30), vitamin D from dairy foods (in quintiles), supplemental vitamin D (ie, 0, >0 to <600, or ≥600 IU/d), OC use (ie, never, past, or current), duration of OC (ie, never, 1-23, 24-47, 48-71, 72-95, 96-119, ≥120 months, or missing), and infertility because of ovulatory disorder (ie, yes or no).

^c^ Additionally adjusted for parity (ie, 0, 1, 2, 3, or ≥4).

To further evaluate whether associations of early menopause and breastfeeding were independent of parity, we stratified our population by parity and evaluated associations separately within groups of women with 1, 2, and 3 or more full-term pregnancies (Figure). Within each stratum, risk was lowest among those reporting exclusive breastfeeding for approximately 7 to 12 months compared with less than 1 month (eg, women with 2 pregnancies: HR, 0.79; 95% CI, 0.66-0.96; women with ≥3 pregnancies: HR, 0.68; 95% CI, 0.52-0.88). In these analyses, risk was not significantly lower among those with cumulative duration of exclusive breastfeeding longer than 12 months (eg, women with 2 pregnancies and duration ≥13 months: HR, 0.87; 95% CI, 0.66-1.15; women with ≥3 pregnancies and duration 13-18 months: HR, 0.86; 95% CI, 0.66-1.13; women with ≥3 pregnancies and duration ≥19 months: HR, 0.98; 95% CI, 0.72-1.32).

**Figure.**
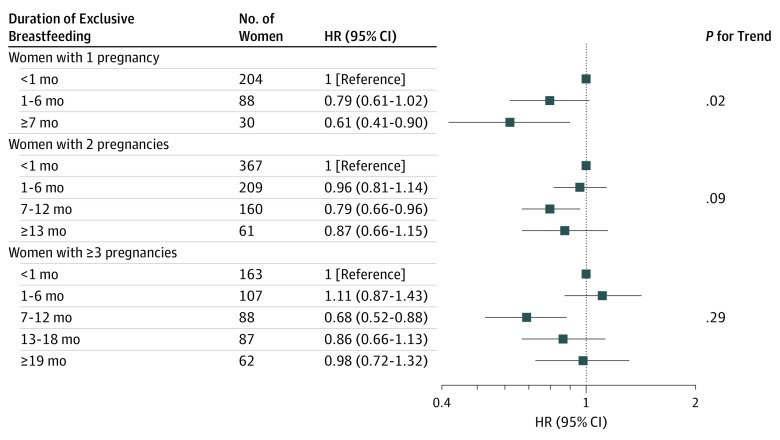
Multivariable Associations of Duration of Exclusive Breastfeeding Stratified by Parity in the Nurses’ Health Study II, 1989-2015 Exclusive breastfeeding was defined as when the infant received breast milk only, with no other liquids or solids given. Parity was defined as pregnancies lasting 6 or more months. Data were from 59 388 parous women who provided information on exclusive breastfeeding. HR indicates hazard ratio.

Results from sensitivity analyses adjusting for HT use and censoring follow-up at first HT use were essentially identical to the main findings (data not shown). In fully adjusted models accounting for parity and breastfeeding, age at first birth was not associated with early menopause (data not shown). Lastly, results comparing parity at ages 25, 30, and 35 years were similar to the main analysis and did not suggest differences by age (data not shown).

## Discussion

To our knowledge, this is the largest study examining the association of reproductive factors with early menopause to date. We observed a significant inverse association of parity with risk of early menopause. Among parous women, total breastfeeding was also associated with a significantly lower risk of early menopause independent of parity. In particular, women who breastfed exclusively for a total of 7 to 12 months during their premenopausal years experienced the lowest risk.

To date, only 1 other study^32^ has examined the association of parity with risk of early natural menopause. Using pooled data from the International Collaboration for a Life Course Approach to Reproductive Health and Chronic Disease Events (InterLACE) multicountry women’s health study, Mishra et al^32^ examined the risk of early menopause among 51 450 postmenopausal women using a multinomial logistic regression model with 6 categories of age at menopause (ie, <40, 40-44, 45-49, 50-51, 52-53, and ≥54 years). Menopause occurring at younger than 40 years was defined as premature menopause, and menopause between the ages 40 and 44 years was considered early menopause. Mishra et al^32^ observed that, after mutual adjustment for parity and age at menarche and adjustment for confounders, nulliparous women were at increased risk for premature menopause, early menopause, and menopause at age 45 to 49 years. Our findings in the NHS2 were similar to those of Mishra et al^32^ in that we observed nulliparous women to be at an increased risk of menopause at younger than 45 years. Similar to the study by Mishra et al,^32^ we also adjusted for body mass index and smoking status; however, our findings were attenuated after further adjustment for infertility status, dietary factors, OC use, and breastfeeding. The present study notably extends the work of Mishra et al^32^ by using prospective reproductive data, accounting for time-varying confounding in Cox models, and considering the contribution of breastfeeding to observed associations of parity with early menopause.

To our knowledge, ours is the first prospective cohort study to examine the association of breastfeeding with the risk of early menopause. We observed that breastfeeding was associated with a lower risk of early menopause independent of parity, with the suggestion of a nonlinear association. Previous studies examining the association of breastfeeding with menopause timing have primarily been cross-sectional and limited by small sample size.^16,17,18,21,30,34,35^ We collected detailed information on breastfeeding and the timing of the introduction of supplemental food, allowing us to estimate the duration of exclusive as well as total breastfeeding. We hypothesized that exclusive breastfeeding would be more strongly associated with reduced risk of early menopause than total breastfeeding because exclusive breastfeeding is more likely to suppress ovulation and thus slow the depletion of the ovarian pool.^7,38^ When breastfeeding is frequent and higher volumes of breast milk are produced, as during exclusive breastfeeding, the secretion of follicle-stimulating hormone and luteinizing hormone are inhibited, while the secretion of the hormone prolactin is stimulated. The interplay of these fluctuating hormone levels can decrease the responsiveness of the ovaries to follicle-stimulating hormone and lutenizing hormone and inhibit ovulation.^38,39^ While our results did not demonstrate a clear dose-response association or clearly elucidate the biological mechanism activated by longer duration of breastfeeding (ie, ovulation suppression vs slowing the rate of follicular atresia), our findings were consistent with this hypothesis and suggest that the inverse association of exclusive breastfeeding with the risk of early menopause reaches a threshold at somewhere between 6 and 12 months. This time frame is aligned with recommendations to breastfeed exclusively for 6 months to confer optimal benefits to both infant and mother.^40,41^

### Limitations

Our study has several limitations. Although we relied on self-report of the onset of menopause, which might contribute to some misclassification,^42,43^ we collected menopause data prospectively. Furthermore, self-assessment of menopause was validated in the Nurses’ Health Study, in which 82% of women repeatedly recalled the same age at menopause within 1 year on repeated questionnaires and age at menopause was confirmed via medical records for 99% of women.^44^ We also relied on self-report of breastfeeding history and duration collected in a prospective manner. Validation studies have demonstrated short-term and long-term recall of breastfeeding to be fairly accurate.^45,46^ Given the prospective design of our study, misclassification of breastfeeding is expected to be nondifferential with respect to menopause; therefore, it would be unlikely that our observed associations were because of misclassification. An examination of the number of months before menses returned after delivery instead of months of breastfeeding may have provided a more accurate depiction of absence of ovulation.^47^ Although this question was asked retrospectively on 1 follow-up questionnaire, we did not have sufficient data for a meaningful analysis. Our study population is fairly homogeneous with respect to race and ethnicity, but we would expect that the physiological association between the reproductive factors of parity, breastfeeding, and early menopause would not differ substantially by race/ethnicity. However, additional evaluation of these associations in more diverse populations^48^ as well as further study of the association with anti-Müllerian hormone levels^49,50^ are important.

## Conclusions

In this large, prospective cohort study that included more than 108 000 premenopausal women, we observed significant inverse associations of both parity and breastfeeding with the risk of early natural menopause. Our findings suggest that some of the lower risk of early natural menopause attributed to parity could be attributable to breastfeeding. Our results suggest that breastfeeding at levels consistent with current recommendations may confer an additional benefit of lower risk of early natural menopause.
